# Novel application of sodium manganese oxide in removing acidic gases in ambient conditions

**DOI:** 10.1038/s41598-023-29274-9

**Published:** 2023-02-09

**Authors:** Nishesh Kumar Gupta, Srungarpu N. Achary, Herlys Viltres, Jiyeol Bae, Kwang Soo Kim

**Affiliations:** 1 Department of Environmental Research, University of Science and Technology (UST), Daejeon, 34113 Korea; 2 Department of Environmental Research, Korea Institute of Civil Engineering and Building Technology (KICT), Goyang, 10223 Korea; 3 Homi Bhabha National Institute, Anushaktinagar, Mumbai 400085 India; 4 Chemistry Division, Bhabha Atomic Research Centre, Trombay, Mumbai 400085 India; 5 School of Engineering Practice and Technology, McMaster University, 1280 Main Street, West Hamilton, ON L8S 4L8 Canada

**Keywords:** Green chemistry, Materials chemistry

## Abstract

In this study, we have demonstrated the application of sodium manganese oxide for the chemisorption of toxic acidic gases at room temperature. The fabricated alkali ceramic has Na_0.4_MnO_2_, Na_2_Mn_3_O_7_, and Na_x_MnO_2_ phases with a surface area of 2.6 m^2^ g^–1^. Na-Mn oxide was studied for oxidation of H_2_S, SO_2_, and NO_2_ gases in the concentration range of 100–500 ppm. The material exhibited a high uptake capacity of 7.13, 0.75, and 0.53 mmol g^–1^ for H_2_S, SO_2_, and NO_2_ in wet conditions, respectively. The material was reusable when regenerated simply by soaking the spent oxide in a NaOH-H_2_O_2_ solution. While the H_2_S chemisorption process was accompanied by sulfide, sulfur, and sulfate formation, the SO_2_ chemisorption process yielded only sulfate ions. The NO_2_ chemisorption process was accomplished by its conversion to nitrite and nitrate ions. Thus, the present work is one of the first reports on alkali ceramic utilization for room-temperature mineralization of acidic gases.

## Introduction

The unprecedented rise in global energy requirements has triggered a massive climate imbalance around the globe. The emission from the combustion of fossil fuels (coal, petroleum, and natural gas) is the main reason behind the increasing concentration of acidic gases in the atmosphere. As per the study published in “The Lancet Planetary Health,” nearly 3 million global deaths in 2019 were associated with air pollution, making it one of the leading causes of death^1^. Moreover, these air pollutants are responsible for soil and water sources acidification, infrastructure corrosion, and loss of biodiversity^2^. Since it is impossible to switch from fossil fuels to green energy alternatives overnight, developing cleaner and more effective air decontamination technologies is more appropriate. These technologies are expected to clean fuel gases before combustion and flue gases originating from thermal power plants and industries.

The adsorption of acidic gases over porous materials like activated carbon^3,4^ or metal–organic frameworks (MOFs)^5,6^ is a go-to-response in the research community due to the availability of a large surface area for the gas to interact and the possibility of tuning these materials for better gas capture. MOFs exhibit a large surface area and specific interactions with acidic gas molecules, making them highly effective in gas capture. However, these materials are expensive and use toxic chemicals in their preparation^7^. Besides, these MOFs perform poorly at a low gas concentration (100–1000 ppm), making their role debatable in deep flue gas desulfurization and natural gas purification. The best MOF that has reached these requirements is Co-gallate, which could adsorb SO_2_ at a low pressure of 0.002 bar at 298 K^8^.

Previously, we developed MOF-derived Na-Mn oxides for room-temperature H_2_S mineralization at an ultra-low pressure of 0.0005 bar, where the derived oxide had a maximum adsorption capacity of 24.1 mmol g^–1^ with partial regenerability^9^. However, the method developed for synthesizing Na-Mn oxide was still expensive and involved toxic chemicals. To overcome these challenges, we have adopted a low-cost and eco-friendly approach to fabricating Na-Mn oxide, specifically, Na_*x*_MnO_2_. Na-Mn oxides in rechargeable Na-ion batteries are among the most explored materials for energy storage applications^10–12^. Other than that, these materials have found applications in radionuclides sequestration^13,14^ and heterogeneous catalysis^15,16^. However, these alkali ceramics are rarely explored for air decontamination applications, though these have better prospects of eliminating acid gases even at room temperature.

In this study, we have exploited the basic sites in Na_*x*_MnO_2_ for room-temperature oxidation of H_2_S, SO_2_, and NO_2_ gases in ambient conditions. The fabricated material was tested in column breakthrough configuration with 100–500 ppm of acidic gases to confirm its applicability in real-world problems involving flue gas purification^17,18^. The material exhibited a better gas chemisorption efficiency in wet conditions. The underlying mechanism driving the gas chemisorption process over the alkali ceramic was probed by various microscopic and spectroscopic techniques. The material possessed a high gas uptake behaviour even after three adsorption-regeneration cycles when regenerated by a green and inexpensive solution. We believe that the developed Na-Mn oxide ceramic is an affordable and green solution for tackling the rising concentrations of acidic gases in the atmosphere. With this work, we are opening a newer application of alkali ceramics in air decontamination applications.

## Methods

### Chemicals

Manganese(II) acetate tetrahydrate (Mn(CH_3_COO)_2_·4H_2_O) and Sodium acetate trihydrate (CH_3_COONa·3H_2_O) were procured from Samchun Pure Chemicals, Korea. Sodium hydroxide (2 mol L^–1^ NaOH) and hydrogen peroxide (28.0 Vol.% H_2_O_2_) solution were procured from Daejung Chemicals and Metals Co. Ltd, Korea. Highly pure H_2_S (Vol. 0.05%), SO_2_ (Vol. 0.01%), and NO_2_ (Vol. 0.01%) gas in N_2_ gas were procured from Union gas, Korea. All the chemicals were of analytical grade and used without any further purification.

### Fabrication of Na_x_MnO_2_

Mn(CH_3_COO)_2_·4H_2_O (12.25 g) and CH_3_COONa·3H_2_O (3.40 g) in a molar ratio of 2:1 were dissolved in a minimum volume of deionized water. The solution was dried at 130 °C overnight (paste-like composition) and pre-calcined at 300 °C for 2 h in a muffle furnace for acetate decomposition. The black powder was further calcined at 700 °C for 8 h to yield the resultant Na_*x*_MnO_2_ abbreviated as NMO.

### Analytical instruments

The oxide morphology was probed through field emission scanning electron microscopy (FE-SEM, Hitachi S-4300, Hitachi, Japan) and field emission transmission electron microscopy (FE-TEM, JEM-2010F, JEOL Ltd., Japan). Elemental mapping was conducted using energy-dispersive X-ray spectroscopy (EDAX) (X-Maxn 80T, Oxford Instruments, United Kingdom) in TEM mode. The X-ray diffraction patterns were recorded at 298 °C between 2θ = 5–100°) on an Ultima IV (Rigaku, Japan) X-ray diffractometer with Cu Kα radiation (λ = 1.5406 Å) and a Ni filter. Fourier transform infrared (FT-IR) spectra were collected over a Cary670 FTIR spectrometer (Agilent Technologies, United States). The specific surface area and porosity of samples were determined by analysing the standard N_2_ adsorption–desorption isotherm at − 196 °C using a Gemini 2360 series (Micromeritics, United States) instrument after degassing at 150 °C for 8 h. X-ray photoelectron spectroscopy (XPS: Nexsa X-Ray Photoelectron Spectrometer System, Thermo Scientific, United Kingdom) was used to determine the chemical states of the elements in the samples. A monochromatic Al Kα X-ray source was used with a fixed pressure of 4.8 × 10^−9^ mbar. Spectra were charge corrected to the main line of the C 1s spectrum (aromatic carbon) set to 284.7 eV. Spectra were analysed using CasaXPS software (version 2.3.14) with GL(*p*) = Gaussian/Lorentzian product formula, where the mixing is determined by *m* = *p*/100, GL(100) is a pure Lorentzian, while GL(0) is a pure Gaussian. We used GL(30).

### Breakthrough experiments

A 250 mg of the adsorbent was placed in a Pyrex tube between glass wool, and a gas flow of 200 mL min^−1^ was maintained. The samples were fully saturated with moisture by blowing water vapour (80% relative humidity) at 25 °C for 20 min through the adsorbent bed. The H_2_S concentration in the outflow gas was analysed by an H_2_S gas analyser (GSR-310, Sensoronic, Korea). The SO_2_ concentration in the effluent gas was analysed using GASTIGER 6000 SO_2_ analyser (Wandi, Korea). The material was tested for NO_2_ adsorption with a flow rate of 100 mL min^−1^. The NO_2_ concentration was analysed using GASTIGER 6000 NO_2_ analyser (Wandi, Korea). The adsorption capacity (*q*, mmol g^−1^) at the breakthrough point (20% of the input gas) was calculated by the following equation:

1 $$q=\frac{{C}_{0}Q}{m{M}_{w,g}}{\int }_{0}^{{t}_{b}}\left(1-\frac{C}{{C}_{0}}\right)dt$$

*C*_0_—initial concentration, *Q*—flow rate, *m*—the mass of oxide (g), *M*_w,g_—gas molecular weight, and *t*_b_—breakthrough time.

Regeneration of spent oxide was done by soaking it in a binary solution (10 mL) of 0.25 mol L^−1^ of NaOH and 0.50 mol L^−1^ of H_2_O_2_ for 8 h. After phase separation, the material was dried and studied for gas adsorption experiments. The same solution was reused for the subsequent regeneration cycles. A mass loss during the regeneration process was expected, and normalized time was adopted to calculate the adsorption capacity.

## Results and discussion

### Characterization of Na_x_MnO_2_

**Figure 1:**
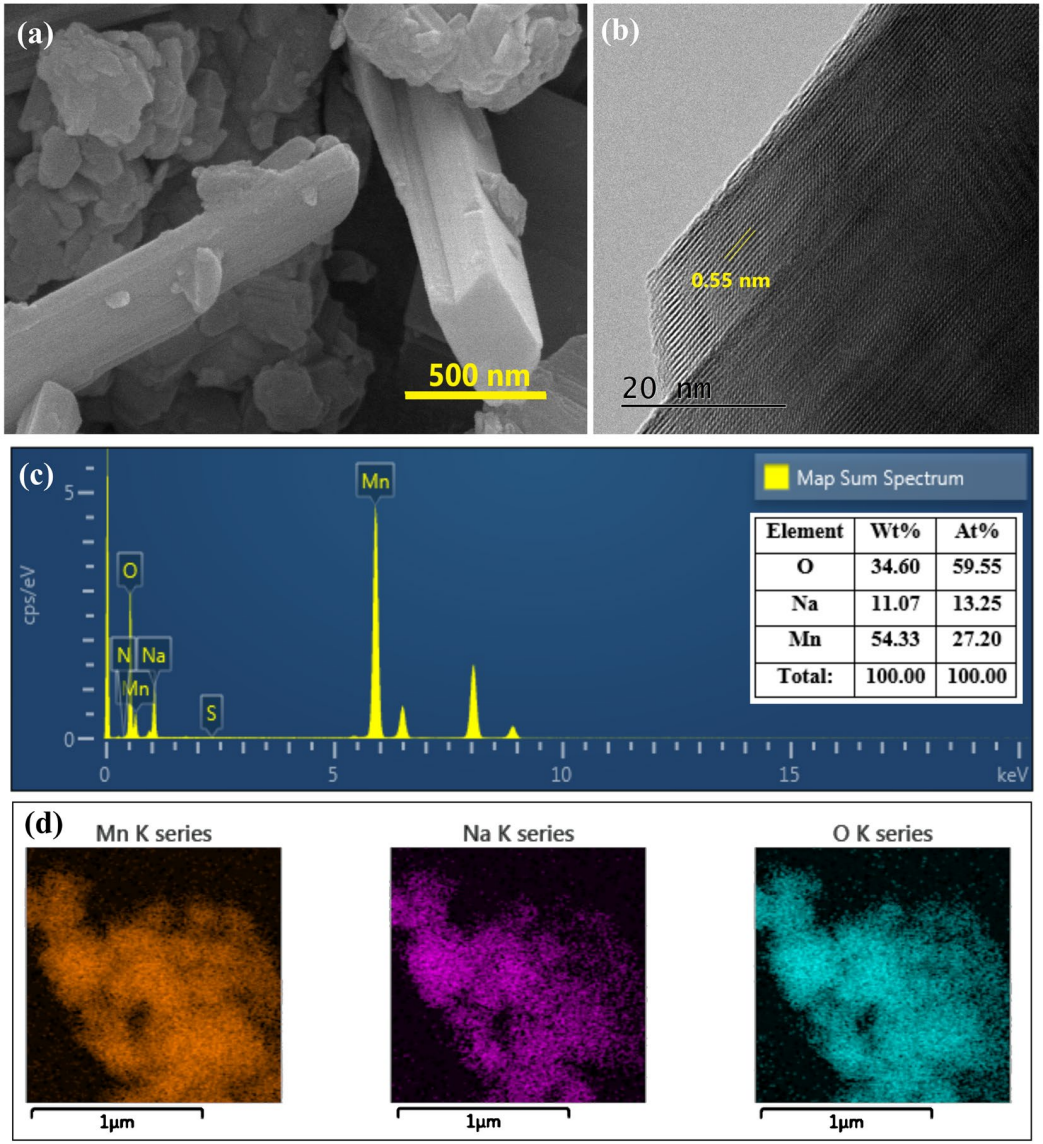
(**a**) SEM micrograph; (**b**) HR-TEM micrograph; (**c**) EDAX analysis; (**d**) 2D elemental mapping of NMO.

The SEM micrograph of NMO confirmed two different morphologies, a microrod-like feature (for Na_0.4_MnO_2_)^19^ and irregularly shaped microsheets (for Na_2_Mn_3_O_7_ and birnessite)^20^, for the synthesized material. The presence of different morphologies was due to the formation of different phases of Na_*x*_MnO_2_ (Fig. 1a)^21^. In the HR-TEM image, the microrod exhibited a fringe width of 0.55 nm corresponding to the 201 reflection of Na_0.4_MnO_2_, which further confirmed that the microrod feature was for the Na_0.4_MnO_2_ phase (Fig. 1b)^19^. The EDAX analysis confirmed the peaks for Mn, Na, and O at respective energy. Moreover, the Na: Mn ratio of 0.49 was nearly equal to the initial ratio of 0.5 for the metal precursors (Fig. 1c). The 2D elemental mapping showed a low and irregular distribution of ‘Na’ over the ‘Mn’ map, probably due to a low Na: Mn ratio in Na_0.4_MnO_2_ and a comparatively higher ratio of 0.7 in Na_2_Mn_3_O_7_ (Fig. 1d).

**Figure 2:**
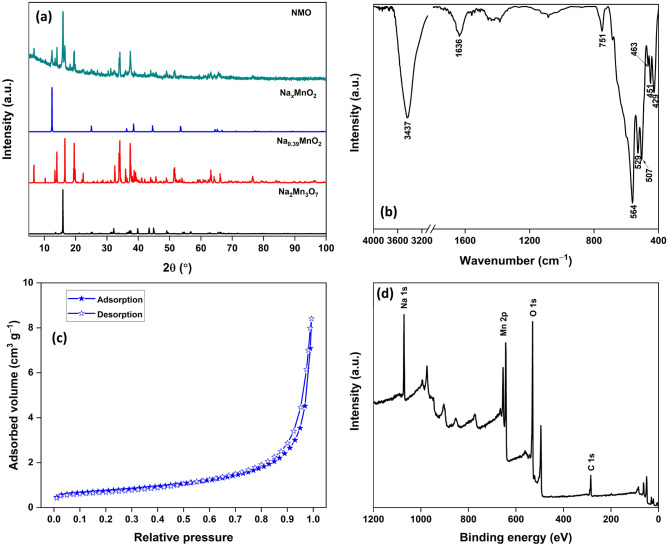
(**a**) PXRD pattern; (**b**) FTIR spectrum; (**c**) N_2_ adsorption–desorption isotherm; (**d**) XPS survey of NMO.

The Rietveld refinement of the XRD data was done to deduce the phases and structures in the prepared NMO sample. The observed and calculated diffraction patterns were in close agreement. The typical Rietveld refinement plot (Figure S1) and the refined structural parameters (Table S1) are available in the ESI file. The PXRD pattern of NMO and identified phases are presented in Fig. 2a. The first major phase was identified as Na_0.4_MnO_2_, crystallized in an orthorhombic lattice and space group *Pbnm* (*a* = 9.0741 Å, *b* = 26.4304 Å, *c* = 2.8240 Å)^22^. The second major phase was triclinic Na_2_Mn_3_O_7_ crystallized in P-1 space group (*a* = 6.604 Å, *b* = 6.851 Å, *c* = 7.527 Å, *α* = 106.29°, *β* = 106.63°, *γ* = 111.65°)^23,24^. A minor phase was identified as rhombohedral Na_*x*_MnO_2_ (Birnessite) crystallized in the *P*-3 space group (*a* = 2.8583 Å, *c* = 7.1108 Å)^25^. Thus, the analysis validated the presence of different Na-Mn oxide phases in the NMO sample.

The FTIR spectrum of NMO has a sharp high-intensity band at 3437 cm^−1^, which was assigned to the O–H stretching vibration of –OH/H_2_O. A low-intensity band at 1636 cm^−1^ was associated with the bending mode of adsorbed H_2_O molecules. The bands at 1449 and 751 cm^−1^ were attributed to the stretching Mn–OH and Na–O vibrations, respectively. Multiple sharp bands between 400–470 and 500–570 cm^−1^ were associated with the stretching vibrations of Mn–O/Na–O (Fig. 2b)^9,26^. The surface and pore characteristics of NMO were evaluated by N_2_ adsorption–desorption isotherms (Fig. 2c). The oxide exhibited type II isotherm behavior, generally observed for nonporous or macroporous materials^27^. The average pore diameter (*D*_p_ ~ 18 nm) and pore volume (*V*_p_ ~ 0.01 cm^3^ g^–1^) were calculated using the Brunauer–Emmett–Teller (BET) and Barrett–Joyner–Halenda method, respectively (Table S2). The surface area of 2.6 m^2^ g^–1^ was estimated using the BET method. The XPS survey of NMO has peaks for Na 1s, Mn 2p, and O 1s at respective binding energy. The C 1s peak was due to the adventitious carbon contamination (Fig. 2d)^28^.

**Figure 3:**
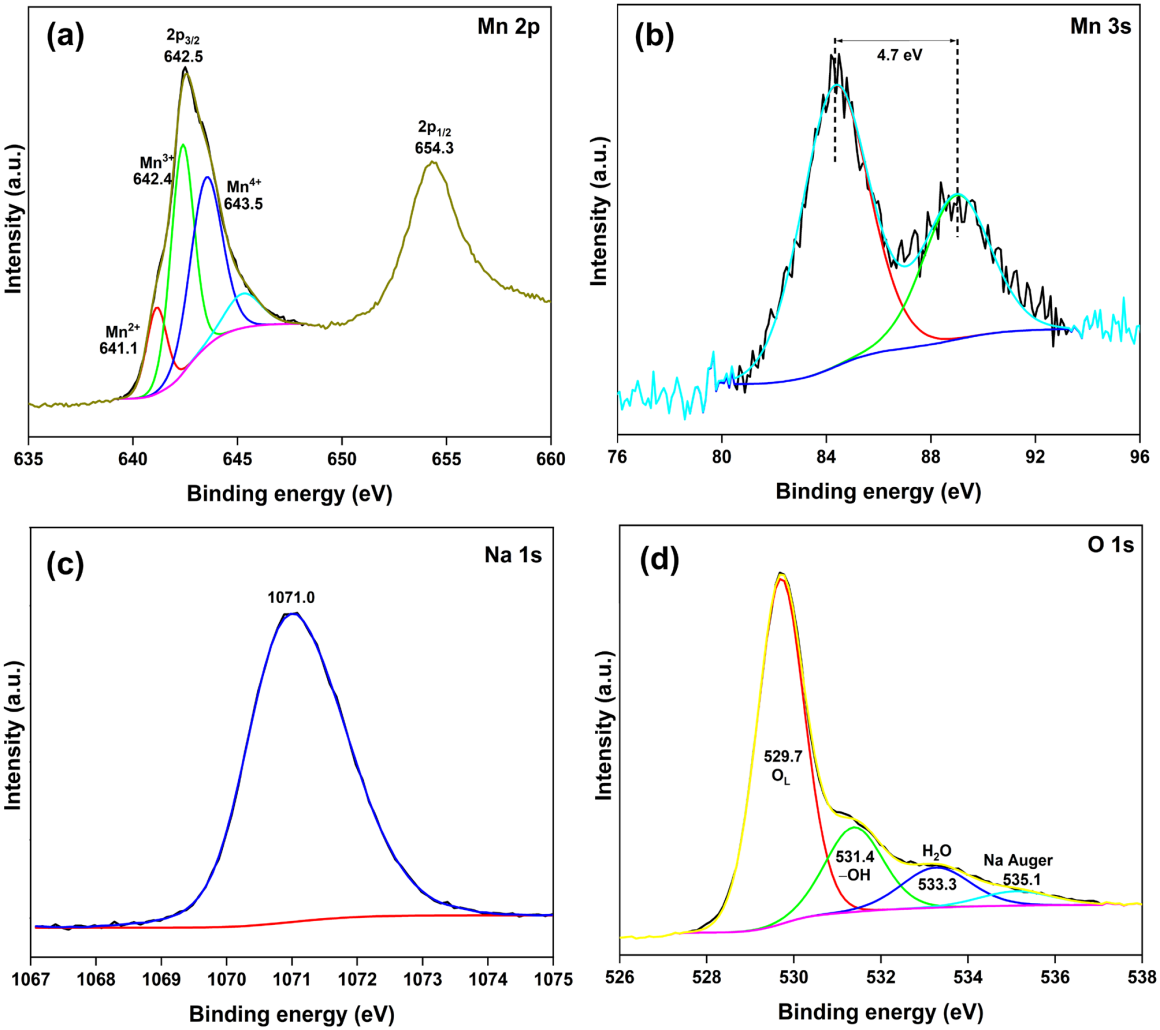
HRXPS (**a**) Mn 2p; (**b**) Mn 3s; (**c**) Na 1s; (**d**) O 1s spectra of NMO.

The HRXPS Mn 2p spectrum of NMO has two peaks at 642.5 and 654.3 eV for 2p_3/2_ and 2p_1/2_, respectively (Fig. 3a, Table S3). The Mn 2p_3/2_ peak was deconvoluted into three contributions at 641.1 (14.1%), 642.4 (42.6%), and 643.5 eV (43.2%), which were assigned to the Mn^2+^, Mn^3+^, and Mn^4+^ oxidation states, respectively^29,30^. Previously reported studies on Na_*x*_MnO_2_-type materials have confirmed the existence of Mn ions exclusively in the + 4 and + 3 oxidation states^19,30^. However, the permanent existence of Mn^2+^ ions in these materials is a likely event, which has been observed for NaMnO_2_-type material^31^. Here, we have noticed a minor yet significant fraction of Mn ions in the + 2 oxidation state, following our reported study on NaMn_*x*_O_*y*_, where the oxide possessed 10.2% of Mn in the divalent state^32^. Thus, the analysis confirmed mixed valence states for Mn with an average oxidation state of + 3.3. The HRXPS Mn 3s spectrum could also shed light on the oxidation states of Mn in the material. The Mn 3s spectrum displayed two peaks at 84.3 and 89.0 eV with an energy separation value of 4.7 eV, which further indicated a major presence of Mn in the + 3 and + 4 oxidation states (Fig. 3b, Table S4)^19^. The HRXPS Na 1s spectrum has one peak at 1071.0 eV, which was associated with Na^+^ ions in the NMO structure (Fig. 3c)^19^. The HRXPS O 1s spectrum was deconvoluted into four contributions at 529.7, 531.4, 533.3, and 535.1 eV for lattice oxygen (O_L_), surface-bound hydroxyl groups (− OH), adsorbed H_2_O^33^, and Na Auger^34^, respectively (Fig. 3d, Table S5).

### Gas breakthrough studies

**Figure 4:**
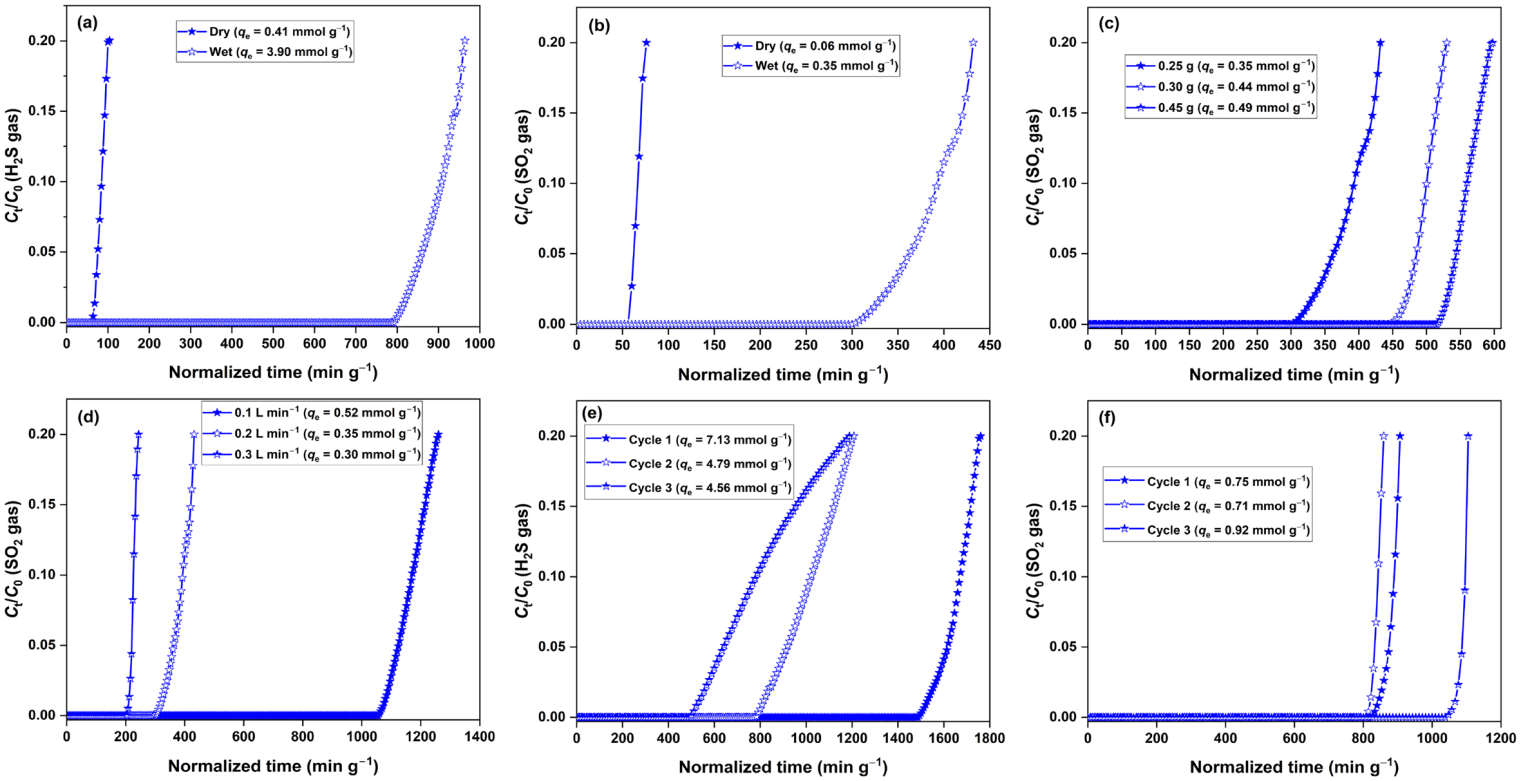
(**a**) H_2_S; (**b**) SO_2_ breakthrough curves for dry/wet NMO; effect of (**c**) adsorbent mass; (**d**) gas flow rate on the SO_2_ adsorption capacity; regeneration efficiency of wet NMO for (**c**) H_2_S; (**d**) SO_2_ adsorption.

The H_2_S and SO_2_ adsorption capacities of NMO were measured in dry and wet conditions (Fig. 4). The adsorption capacity of NMO was 0.41 and 3.90 mmol g^−1^ in dry and wet conditions, respectively (Fig. 4a). The SO_2_ adsorption capacity of NMO was 0.06 and 0.35 mmol g^−1^ in dry and wet conditions, respectively (Fig. 4b). These many-fold improvements in the adsorption capacity upon water vapour addition in the adsorbent bed suggested a strong involvement of the acidic gas dissolution process during the process. The dissolution of H_2_S ${(\mathrm{H}}_{2}\mathrm{S}(g)+{\mathrm{H}}_{2}\mathrm{O}(l)\to {\mathrm{HS}}^{-}(l)+{\mathrm{H}}_{3}{\mathrm{O}}^{+}(l))$^35^ or SO_2_ (${\mathrm{SO}}_{2}(g)+{\mathrm{H}}_{2}\mathrm{O}(l)\to {2\mathrm{H}}^{+}(l)+{\mathrm{SO}}_{3}^{2-}(l))$^36^ in H_2_O layers to form reactive species is primarily responsible for a high H_2_S/SO_2_ uptake in wet oxides. The oxidation of sulfur (S^0^) and sulfide (S^2−^) species to sulfate (SO_4_^2−^) in the presence of H_2_O and molecular O_2_ further enhances the H_2_S uptake capacity^37^. A similar effect is observed for SO_2_ adsorption, where the introduction of water to the adsorption system favours the SO_2_ uptake process by converting surface oxidized SO_2_ form (i.e., SO_3_) to sulfuric acid^36^. Water molecule dissociative adsorption over the metal oxide surface (to form –OH groups) improves the gas molecule interaction through reactive pathways^35^. Long et al. have reported significant improvement in the H_2_S and SO_2_ uptake gas over Na-MnO_*x*_ xerogel and aerogel in humid conditions^38^. Even in our previously reported study on H_2_S adsorption over NaMn_*x*_O_*y*_, we witnessed ~ 5–6 fold improvement in the gas adsorption capacity when the adsorbent bed was saturated with water vapours^9^. Besides H_2_S and SO_2_, the NMO adsorbent was tested for NO_2_ gas as well (Figure S2). The oxide exhibited an uptake capacity of 0.53 mmol g^−1^ for NO_2_ gas, making it applicable to a broad spectrum of acidic gases.

In the literature, multiple Mn-based adsorbents have been reported to capture acidic gases in ambient conditions (Table 1). The Na-Mn oxide composite fabricated in this study outperforms many previously reported materials on efficacy and regenerability. However, some materials like Na-MnO_*x*_ aerogel have demonstrated exceptionally high adsorption capacity with no regenerability^38^. Even MOF-derived NaMn_*x*_O_*y*_ has shown a record-breaking capacity of 24.1 mmol g^−1^^9^. However, these studies involved template-based synthesis approaches, which are tedious, energy-intensive, and environmentally unfriendly. Though adsorbent like Mn_3_O_4_/γ-Al_2_O_3_ has shown a high NO_2_ adsorption capacity^39^, we have demonstrated that Na-Mn oxide developed in this study could capture multiple acidic gases at low concentrations of 100–500 ppm.

**Table 1:** The acidic gas adsorption capacity of Mn-based adsorbents at room temperature.

Mn-based adsorbent	Experimental condition			Initial capacity (*q*_e_, mmol g^−1^)	N^th^ cycle (N) (*q*_e_, mmol g^−1^)
	(Mass, g)	Gas (ppm), flow rate (L min^−1^)	BTP (%)		
MOF-derived NaMn_*x*_O_*y*_^9^	0.10	H_2_S (500), 0.1	80	24.10	3.47 (4)
MOF-derived NaMn_*x*_O_*y*_^32^	0.20	SO_2_ (100), 0.2	2	0.33	N.A.
Na_0.4_MnO_2_^47^	0.15	H_2_S (500), 0.2	20	5.29	7.46 (3)
	0.15	SO_2_ (100), 0.2	20	0.87	1.03 (3)
Mn_3_O_4_^48^	0.10	H_2_S (300), N.A.	33	0.47	N.A.
Na-MnO_*x*_ aerogel^38^	N.A.	H_2_S (705), 0.02	100	20.0	N.A.
	N.A.	SO_2_ (375), 0.02	100	3.10	N.A.
Mn_0.025_Zn_0.975_O/SiO_2_^49^	0.50	H_2_S (10,000), N.A.	2	1.09	0.98 (10)
Mn–Zn–Fe oxide nanocomposite^50^	0.20	H_2_S (500), 0.2	20	1.31	N.A.
	0.20	SO_2_ (100), 0.2	20	0.49	N.A.
α-MnO_2_^51^	0.04	SO_2_ (1000), N.A.	100	1.23	N.A.
10 wt.% Mn_3_O_4_/γ-Al_2_O_3_^39^	0.05	NO_2_ (500), 1.0	100	5.38	N.A.
Na-Mn oxide [This study]	0.15	H_2_S (500), 0.2	20	7.13	4.56 (3)
	0.15	SO_2_ (100), 0.2	20	0.75	0.92 (3)
	0.25	NO_2_ (100), 0.1	20	0.53	N.A.

N.A., Not available; BTP, Breakthrough point.

The effect of column operational parameters like adsorbent mass and the gas flow rate was studied with SO_2_ as the target pollutant. The breakthrough experiments were carried out by varying the oxide mass between 0.25 and 0.45 g with a constant SO_2_ flow rate of 0.2 L min^−1^ (Fig. 4c). The SO_2_ adsorption capacity improved from 0.35 to 0.49 mmol g^−1^ with an increase in the oxide loading from 0.25 to 0.45 g. The increasing mass (increasing bed height) improves the gas retention time, which provides a better chance for a gas molecule to interact with the active sites. This increased adsorbate-adsorbent interaction enhances the gas adsorption capacity^40^. The effect of gas flow rate was studied between 0.1–0.3 L min^−1^ with a constant adsorbent mass of 0.25 g (Fig. 4d). The SO_2_ uptake capacity significantly dropped from 0.52 to 0.30 mmol g^−1^ with the rising flow rate from 0.1 to 0.3 L min^−1^. This inverse relationship is due to the decrease in the gas retention time and poor mass transfer with the increasing flow rate, which reduces the adsorbate-adsorbent interaction^41^.

One of the challenges for the reactive adsorption of acidic gases over metal oxides in ambient conditions is the regeneration of material for its judicious use. Metal oxide adsorbents are readily deactivated post-acidic gas adsorption and thermally treated at high temperatures for surface activation and subsequent reuse^42–44^. The idea of thermal regeneration severely limits the novelty of the room-temperature adsorption process as it is energy-intensive and forms secondary pollutants like SO_*x*_^45^. We are focused on developing affordable, zero-energy, and green regeneration strategies, which make the entire adsorption process lucrative. Previously, we have demonstrated partial regeneration of spent NaMn_*x*_O_*y*_ by soaking it in an NH_4_OH solution. The material maintained ~ 47% (9.97 mmol g^−1^) of its initial capacity even after the fourth adsorption-regeneration cycle^9^. Even we have used an H_2_O_2_ solution in the partial regeneration of Ag–Cu-MOF post-H_2_S adsorption^46^.

Here, we have used a binary solution of NaOH and H_2_O_2_ for regenerating spent oxide by soaking it for 8 h, which is green, low-cost, and energy-free. We have demonstrated the applicability of the regeneration process for three cycles for H_2_S and SO_2_. The oxide exhibited an H_2_S adsorption capacity of 7.13 mmol g^−1^ in the first cycle, which dropped to 4.56 mmol g^−1^ (64% of initial capacity) in the third cycle (Fig. 4e). The gas removal capacity is still high even after the third cycle, which makes the entire process efficient for H_2_S uptake. The SO_2_ adsorption capacity of 0.75 mmol g^−1^ in the first cycle improved to 0.92 mmol g^−1^ in the third cycle (Fig. 4f). Thus, the alkali ceramic developed in this study is highly regenerative and effective for the chemisorption of various acidic gases.

### Adsorption mechanism

**Figure 5:**
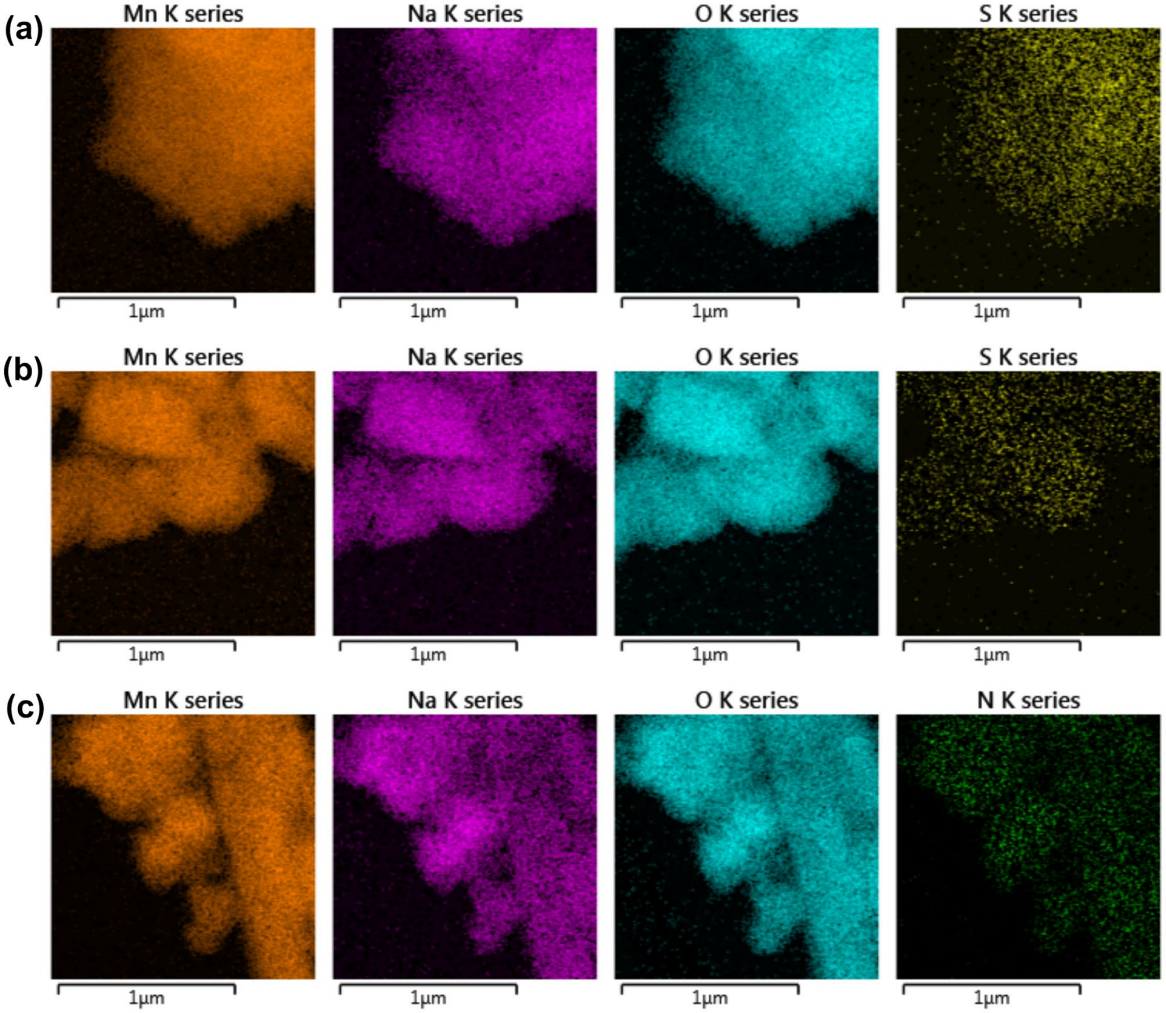
2D elemental mapping of (**a**) NMO_H_2_S; (**b**) NMO_SO_2_; (**c**) NMO_NO_2_.

The SEM micrographs of NMO before and after gas adsorption showed no significant morphological change in the material (Figure S3). The 2D elemental mapping of spent NMO samples is shown in Fig. 5. In all the samples, the ‘Mn’ and ‘Na” density was intact. In H_2_S- and SO_2_-adsorbed NMO samples, wide distribution of ‘S’ was confirmed (Fig. 5a,b). Similarly, for the NO_2_-adsorbed NMO sample, uniform distribution of ‘N’ over NMO was observed (Fig. 5c). The surface area and pore characteristics of water- and gas-exposed NMO samples are available in Table S2. There was no change in the shape of the N_2_ adsorption–desorption isotherms of these samples (Figure S4). The water-exposed-dried sample (hereafter denoted as NMO_H_2_O*) has a higher surface area and pore volume than the pristine sample, which could be one of the many reasons for higher gas adsorption. The surface area and pore volume of the H_2_S-exposed sample were the lowest due to the highest surface coverage with oxidized sulfur species. The Rietveld refinement plot of the NMO_H_2_O* sample remained unaltered with no compositional phases. Moreover, with an insignificant variation in the XRD plots of gas-exposed samples, a surface-driven delocalized chemisorption process was confirmed in this study (Figure S5).

**Figure 6:**
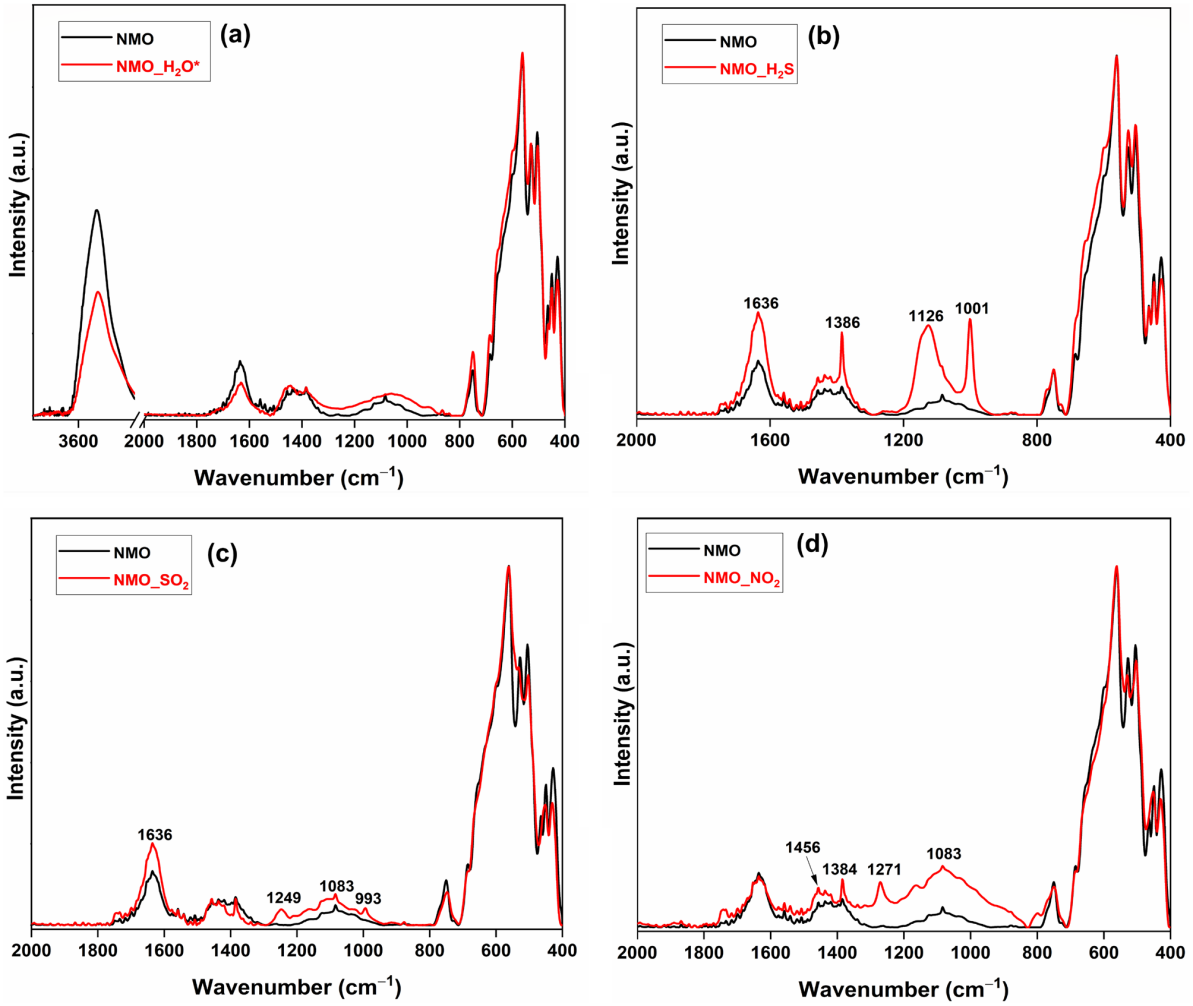
FTIR spectra of (**a**) water-saturated NMO after complete drying; (**b**) NMO_H_2_S; (**c**) NMO_SO_2_; (**d**) NMO_NO_2_.

The FTIR spectra of pristine NMO and NMO_H_2_O* overlapped with all bands intact (Fig. 6a). The FTIR spectrum of the H_2_S-adsorbed sample has new high-intensity bands at 1001 and 1126 cm^−1^ for the stretching vibration *v*_1_ and *v*_3_ modes of SO_4_^2−^ species, respectively (Fig. 6b)^32,52^. For the SO_2_-adsorbed NMO sample, the band at 993 cm^−1^ was related to the stretching vibration of the SO_4_^2−^ species (Fig. 6c). In the FTIR spectrum of NO_2_-adsorbed NMO, bands were observed at 1456, 1384, and 1271 cm^−1^. The band at 1384 cm^−1^ was associated with the stretching vibrations of nitrate (NO_3_^−^) ions. The bands at 1271 and 1456 cm^−1^ were due to the nitrite (NO_2_^−^) ions (Fig. 6d)^53^.

**Figure 7:**
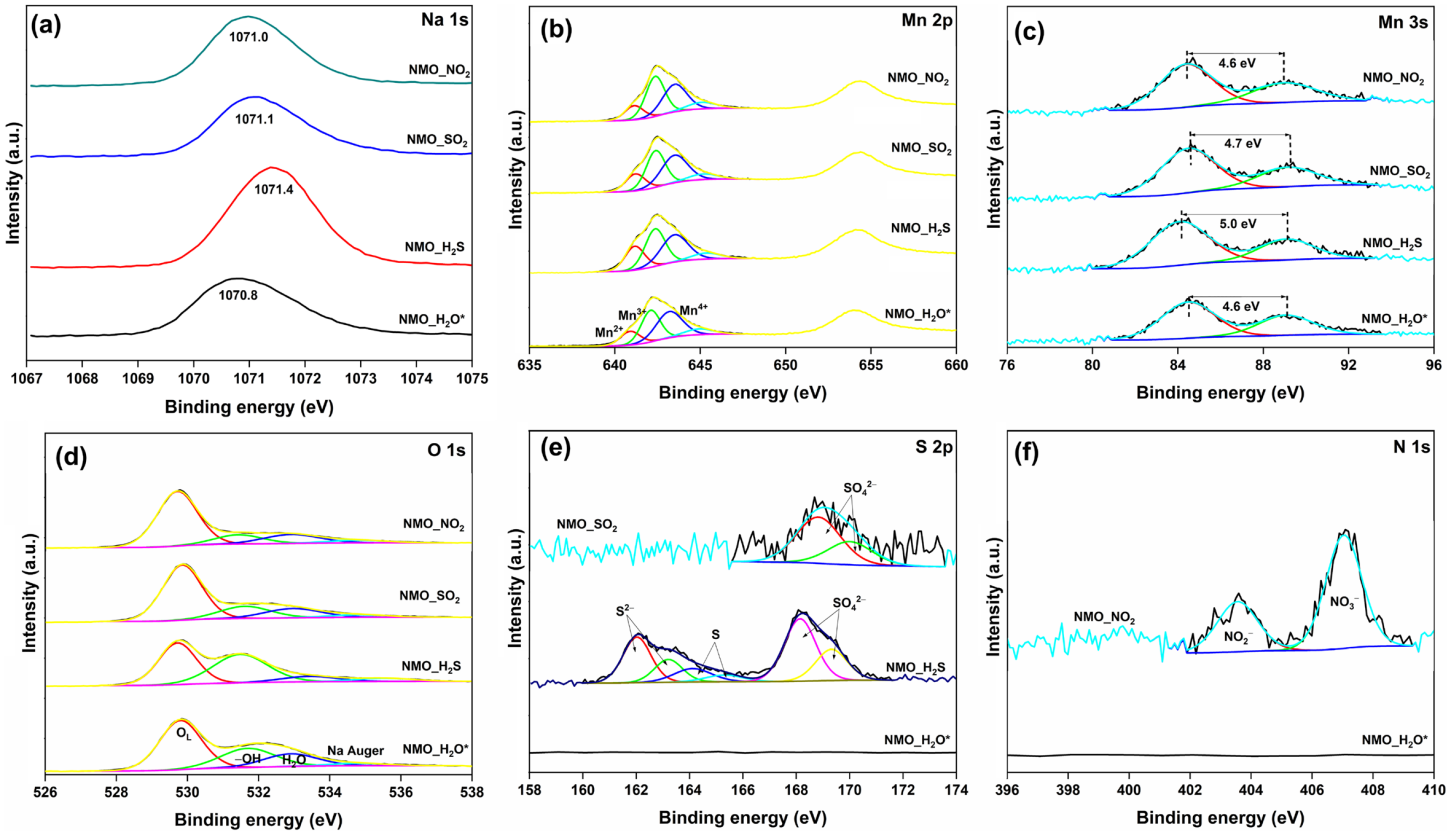
HRXPS (**a**) Na 1s; (**b**) Mn 2p; (**c**) Mn 3s; (**d**) O 1s; (**e**) S 2p; (**f**) N 1s spectra of NMO after acid gas adsorption.

XPS spectroscopy was used to understand the adsorption behavior of NMO for various acidic gases. The peak at 1070.8 eV in the Na 1s spectrum of NMO_H_2_O* redshifted by 0.2 eV compared to the NMO sample. It was possibly due to the interaction of H_2_O molecules with the Na^+^ ions, which increased the electron density in the valence state of Na ions and redshifted the Na 1s peak. The binding energy of Na 1s peak shifted to a higher value upon acidic gas adsorption, which was associated with the removal of H_2_O molecules from Na^+^ ions and the interaction of oxidized sulfur or nitrogen species with the surface Na^+^ ions. This blue shift in the Na 1s binding energy has been witnessed for H_2_S chemisorption over NaMn_0.6_O_3.2_ (Fig. 7a)^9^. The HRXPS Mn 2p spectrum of NMO_H_2_O* has the same three contributions for + 2, + 3, and + 4 oxidation states, with their binding energy shifted to a lower value by 0.2–0.3 eV due to the interaction of H_2_O molecules and the formation of surface –OH groups (Fig. 7b)^54^. The surface hydroxyl formation was confirmed by the increased proportion of 531.7 eV peak in the O 1s spectrum of NMO_H_2_O*. This peak is associated with the hydroxyl group, which improved by 7.3% upon H_2_O exposure (Fig. 7d). This additional formation of Mn-anchored OH groups could serve as the binding sites and oxidation sites for acidic gas molecules^9,32,55^. In the HRXPS Mn 2p spectra of gas-exposed NMO samples, the binding energy for peaks corresponding to all three oxidation states reverted to the value of the fresh NMO sample. For NMO_H_2_S, the Mn^2+^ proportion increased to 24.0 from 17.4% in NMO_H_2_O*. The increased Mn^2+^/Mn^4+^ ratio upon H_2_S adsorption confirmed that Mn redox cycles oxidized S^2−^ to S^0^ with the reduction of Mn^4+^ to Mn^2+^^9,56^. For the NMO_SO_2_ sample, the Mn^2+^ and Mn^3+^ proportion improved at the expense of Mn^4+^ ions. Previously, Quesne-Turin et al.reported the surface oxidation of SO_2_ gas molecules over Li_2_MnO_3_, where the gas probe molecule was oxidized to SO_4_^2−^ and a fraction of Mn^4+^ ions were reduced to Mn^3+^^57^. Here, the SO_2_ oxidation mechanism lowered the Mn oxidation state and formed SO_4_^2−^ species^36^. Like SO_2_, NO_2_ oxidizes on the NMO surface by reducing the Mn oxidation state. Because of this, the Mn^3+^ contribution increased at the expense of Mn^4+^ for the NMO_NO_2_ sample (Fig. 7b). The increased Mn^2+^ ion contribution in the samples could be identified qualitatively from the energy separation value in their respective Mn 3s spectrum. An increase from 4.6 eV (in NMO_H_2_O*) to 5.0 and 4.7 eV for H_2_S- and SO_2_-adsorbed NMO, respectively, further confirmed the increment in Mn^2+^ proportion. Since we did not observe any improvement in Mn^2+^ contribution for NMO_NO_2_, the energy separation value remained the same (Fig. 7c)^58^. One important thing to note here is that though the oxidation state variation is expected during these redox reactions, the proportional changes in the %Mn^n+^ ratio are not high. The possible reason for this is the role of adsorbed molecular O_2_, which could re-oxidize these low oxidation state Mn ions to a higher valency^36,59^. But, from the Mn 2p and Mn 3s analyses, it is confirmed that the Mn sites in NMO are the active sites for the adsorption and oxidation of acidic gas molecules.

The HRXPS O 1s spectrum could not be exploited for extracting new information as the peak for –OH groups at 531.7 eV in gas-adsorbed NMO samples coupled with the S–O bond contribution (from SO_4_^2−^) at 531.8 eV^60^. The HRXPS S 2p spectrum of the H_2_S-adsorbed NMO sample showed a complex spectral shape, which after deconvolution, has three sets of doublets. The S 2p_3/2_ peak at 162.0, 164.1, and 168.1 eV were assigned to the S^2−^ (34.1%)^61^, S^0^/polysulfide, S_n_^n−^ (13.4%)^62,63^, and SO_4_^2−^ (52.5%) (Table S6)^63^, respectively. Thus, three sulfur species were confirmed as the by-products of the H_2_S chemisorption process. The adsorbed H_2_S molecules on the NMO surface dissociated to form S^2−^ ions after chemically reacting with the O_L_ sites or –OH groups. The S^2−^ ions further oxidized to S^0^/S_n_^n−^ by the Mn^4+^/Mn^3+^/Mn^2+^ redox cycle^64^. Finally, these species were oxidized by surface-adsorbed H_2_O and molecular O_2_ to SO_4_^2−^ ions^41^. The formation of these three sulfur species upon H_2_S adsorption is well-documented for NaM_*x*_O_*y*_-type materials^9,41^. The HRXPS S 2p spectrum of NMO_SO_2_ has only one doublet. The 2p_3/2_ peak at 168.8 eV was associated with SO_4_^2−^ species formed after the reactive adsorption of SO_2_. The adsorbed SO_2_ molecule could either be hydrolyzed in the surface H_2_O or directly react with the O_L_ sites/–OH groups on the NMO surface to yield sulfite (SO_3_^2−^) ions. These SO_3_^2−^ ions readily reacted with the molecular O_2_ on the NMO surface to yield SO_4_^2−^ ions^38^. Previously, the interaction of SO_2_ molecules with the LiMn_2_O_3_ surface has confirmed the preferential formation of SO_4_^2−^ over SO_3_^2−^ in ambient conditions^57,65^. Moreover, our previous work confirmed SO_4_^2−^ species formation over NaMn_*x*_O_*y*_ after low-temperature SO_2_ adsorption (Fig. 7e)^32^. The HRXPS N 1s spectrum of NMO_NO_2_ has two well-defined peaks at 403.5 and 407.0 eV with a contribution of 32.9 and 67.1% (Table S7), which were assigned to NO_2_^−^ and NO_3_^−^ ions, respectively^66^. The direct interaction of NO_2_ with O_L_ sites or a disproportionate reaction between NO_2_ molecules on Mn sites could form NO_3_^−^ species on the NMO surface^67^. The disproportionate products NO_3_^−^ and NO^+^ are located on adjacent Lewis acid (Mn^n+^) and Lewis base (O^2−^) sites, respectively, and formed through an intermolecular electron transfer process^66^. The second product of the disproportionate reaction, i.e., NO^+^, reacts readily with the O_L_ sites to form NO_2_^−^ species^68^. Another possible reaction pathway could be the interaction of NO_2_ molecules with the surface –OH group to form NO_3_^−^ ions (Fig. 7f)^69^.

## Conclusion

In this study, we have synthesized a novel and affordable sodium manganese oxide for the chemisorptive removal of low concentrations of toxic acidic gases at room temperature. The fabricated Na-Mn oxide has orthorhombic Na_0.4_MnO_2_, triclinic Na_2_Mn_3_O_7_, and rhombohedral Na_*x*_MnO_2_. The oxide possessed a surface area of 2.6 m^2^ g^–1^ with a dual rod-sheet morphology. The oxide showed a better acidic gas uptake capacity in wet conditions due to the formation of –OH groups on the NMO surface and the involvement of the gas dissolution process. The Na-Mn oxide exhibited a gas uptake capacity of 7.13, 0.75, and 0.53 mmol g^–1^ for H_2_S, SO_2_, and NO_2_, respectively, in wet conditions. The oxide was regenerable for multiple cycles after soaking in a binary NaOH-H_2_O_2_ solution. Insignificant changes in the PXRD pattern of gas-exposed samples confirmed the delocalized chemisorption process on two–three layers of the oxide surface. The spectroscopic analyses confirmed the formation of S^2−^ (34.1%), S^0^ (13.4%), and SO_4_^2−^ (52.5%) species upon the H_2_S adsorption-oxidation process. While SO_2_ molecules oxidized to surface-bound SO_4_^2−^ ions, the NO_2_ oxidation process formed NO_2_^−^ (32.9%) and NO_3_^−^ (67.1%) ions. These surface reactions were mediated by the lattice oxygen, surface hydroxyl groups, and Mn redox cycles in H_2_O and O_2_ presence. Thus, we have demonstrated Na-Mn oxide as a robust and affordable material for removing lowly concentrated acid gases at room temperature. Moreover, the developed regeneration method could make these materials lucrative for air purification processes.

## Supplementary Information

Supplementary Information.

## Data Availability

The data will be made available by N.K. Gupta (guptan@kict.re.kr) only after a reasonable request.
