# Supplementary material for: Novel application of sodium manganese oxide in removing acidic gases in ambient conditions
Source: Sci Rep. 2023 Feb 9;13:2330. doi: 10.1038/s41598-023-29274-9 (PMC9911640; doi:10.1038/s41598-023-29274-9)
Supplement: Supplementary file 1 — Supplementary Information. [file 41598_2023_29274_MOESM1_ESM.docx]

**Supporting Information for:**

Novel Application of Sodium Manganese Oxide in Removing Acidic Gases in Ambient Conditions

Nishesh Kumar Gupta^1,2^, Srungarpu N. Achary^3,4^, Herlys Viltres^5^, Jiyeol Bae^1,2^, Kwang Soo Kim^1,2*^

^1^Department of Environmental Research, University of Science and Technology (UST), Daejeon 34113, Korea
^2^Department of Environmental Research, Korea Institute of Civil Engineering and Building Technology (KICT), Goyang 10223, Korea

^3^Homi Bhabha National Institute, Anushaktinagar, Mumbai 400085, India

^4^Chemistry Division, Bhabha Atomic Research Centre, Trombay, Mumbai 400085, India

^5^School of Engineering Practice and Technology, McMaster University, 1280 Main Street, West Hamilton, Ontario L8S 4L8, Canada

*[kskim@kict.re.kr](mailto:kskim@kict.re.kr) (K.S. Kim)





**Figure S1.** Rietveld refinement plot of NMO. The recorded pattern is shown with black dots, and the Le Bail fit, and its residual is shown with solid blue and red lines, respectively. Vertical ticks indicate the positions of the Bragg peaks for different phases.


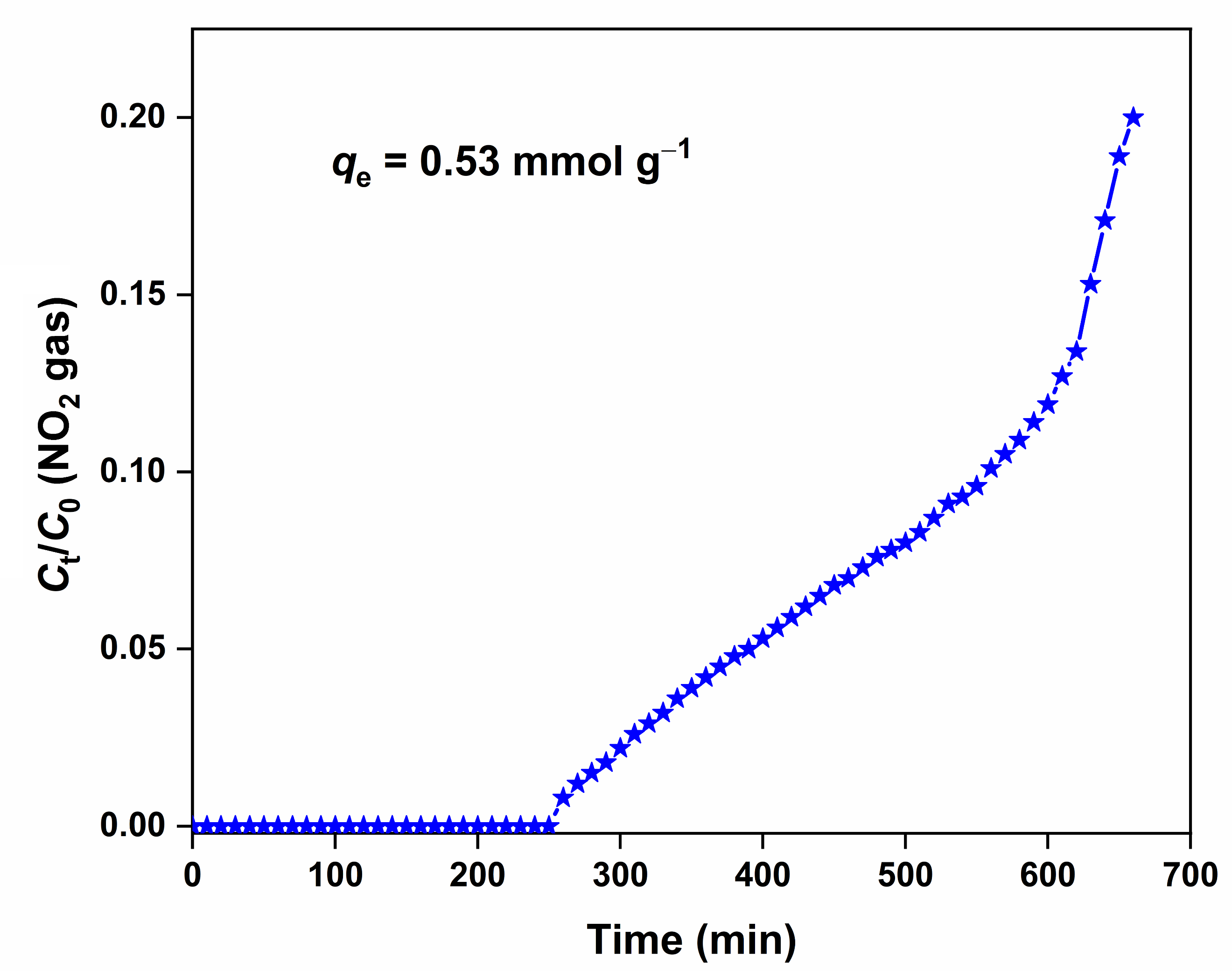


**Figure S2.** NO_2_ breakthrough curve for NMO.


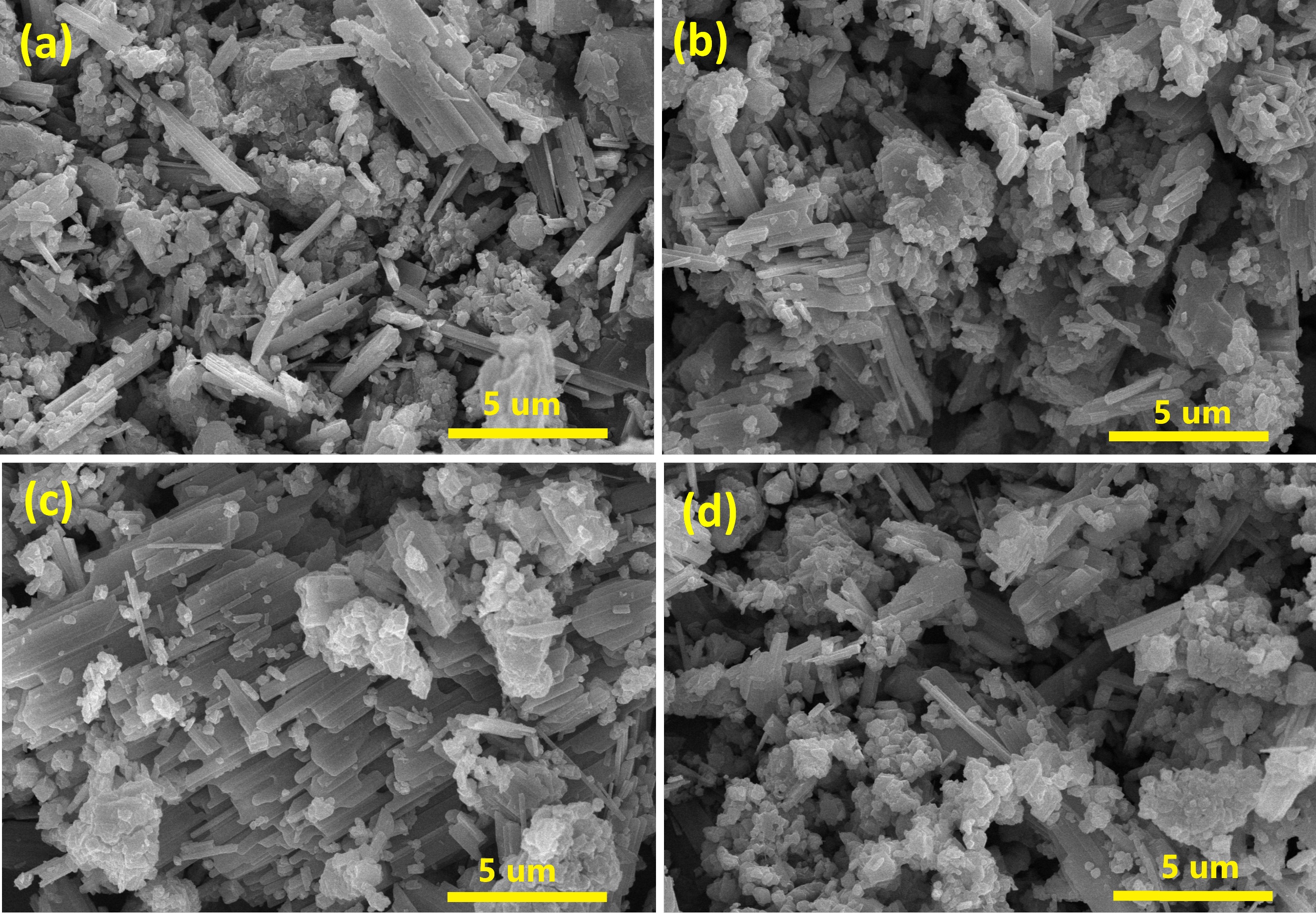


**Figure S3.** SEM micrographs of (**a**) NMO_fresh; (**b**) NMO_H_2_S; (**c**) NMO_SO_2_; (**d**) NMO_NO_2_.





**Figure S4.** N_2_ adsorption-desorption isotherms of water-adsorbed and dried NMO and NMO after acidic gases adsorption.


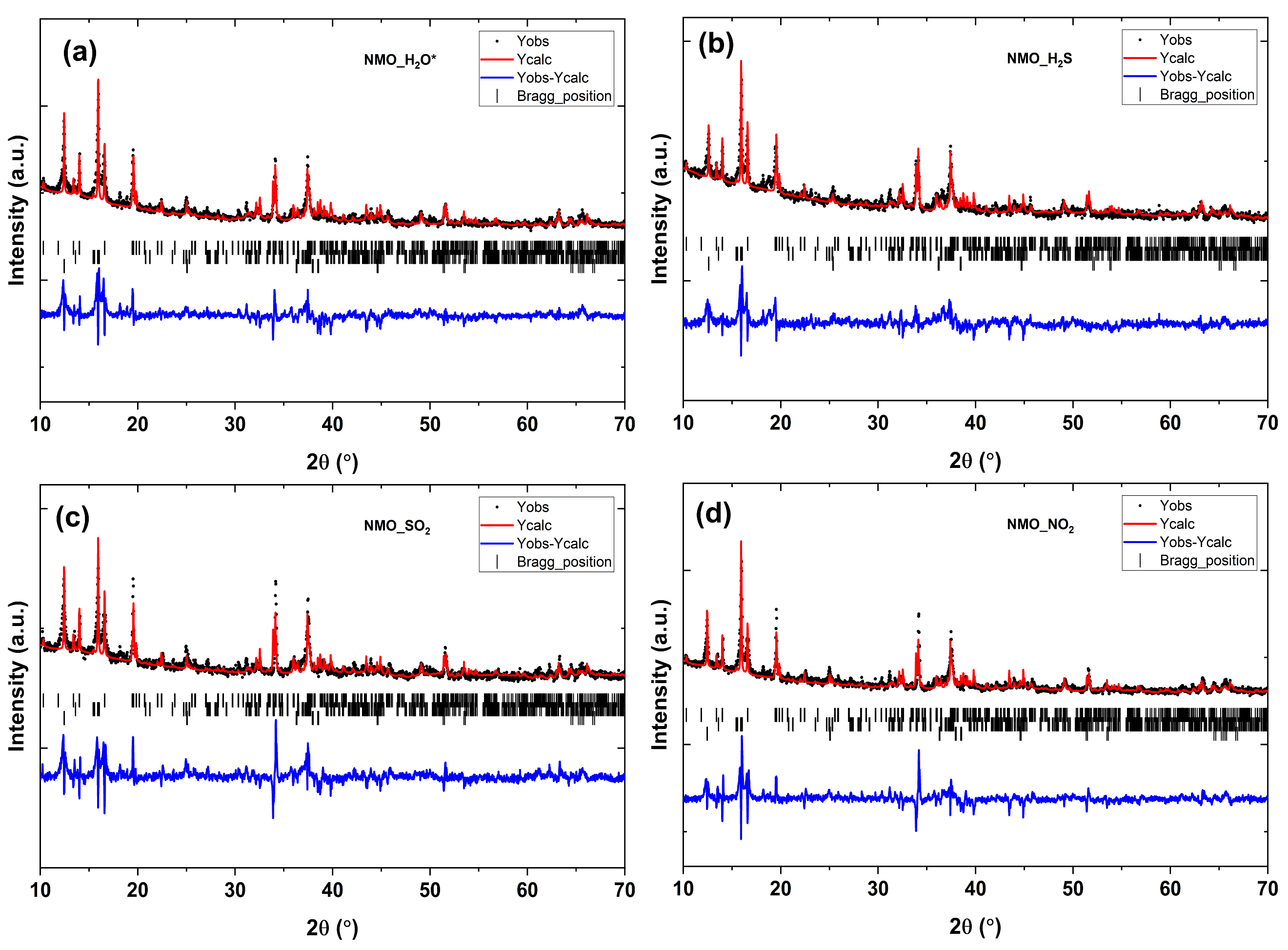


**Figure S5.** Rietveld refinement plot of (**a**) water saturated NMO after complete drying; (**b**) NMO_H_2_S; (**c**) NMO_SO_2_; (**d**) NMO_NO_2_. The recorded pattern is shown with black dots, and the Le Bail fit, and its residual is shown with solid blue and red lines, respectively. Vertical ticks indicate the positions of the Bragg peaks for different phases.

**Table S1.** The refined structural parameters for NMO samples.

| **Phases** | **Na_0.39_MnO_2_**  **Orthorhombic (Pbnm)** | **Na_2_Mn_3_O_7_**  **Triclinic (P-1)** | **Na*_x_*MnO_2_ (Birnessite)**  **Rhombohedral (P-3)** |
| --- | --- | --- | --- |
| **NMO** | *a* = 9.0741 Å  *b* = 26.4304 Å  *c* = 2.8240 Å | *a* = 6.604 Å  *b* = 6.851 Å  *c* = 7.527 Å  *α* = 106.29º  *β* = 106.63º  *γ* = 111.65º | *a* = 2.8583 Å  *c* = 7.1108 Å |
| **NMO_H_2_O*** | *a* = 9.0741 Å  *b* = 26.4304 Å  *c* = 2.8240 Å | *a* = 6.604 Å  *b* = 6.851 Å  *c* = 7.527 Å  *α* = 106.29º  *β* = 106.63º  *γ* = 111.65º | *a* = 2.8583 Å  *c* = 7.1108 Å |
| **NMO_H_2_S** | *a* = 9.0741 Å  *b* = 26.4304 Å  *c* = 2.8240 Å | *a* = 6.604 Å  *b* = 6.851 Å  *c* = 7.527 Å  *α* = 106.29º  *β* = 106.63º  *γ* = 111.65º | *a* = 2.8664 Å  *c* = 7.0258 Å |
| **NMO_SO_2_** | *a* = 9.0741 Å  *b* = 26.4304 Å  *c* = 2.8240 Å | *a* = 6.604 Å  *b* = 6.851 Å  *c* = 7.527 Å  *α* = 106.29º  *β* = 106.63º  *γ* = 111.65º | *a* = 2.8583 Å  *c* = 7.1108 Å |
| **NMO_NO_2_** | *a* = 9.0741 Å  *b* = 26.4304 Å  *c* = 2.8240 Å | *a* = 6.604 Å  *b* = 6.851 Å  *c* = 7.527 Å  *α* = 106.29º  *β* = 106.63º  *γ* = 111.65º | *a* = 2.8583 Å  *c* = 7.1108 Å |

**Table S2.** Surface area and pore characteristics of fresh and gas adsorbed NMO.

| **Sample** | ***S*_BET_ (m^2^ g^−1^)** | ***V*_p_ (cm^3^ g^−1^)** | ***D*_p_ (nm)** |
| --- | --- | --- | --- |
| **NMO_Fresh** | 2.61 | 0.011 | 18.3 |
| **NMO_H_2_O*** | 2.81 | 0.020 | 28.9 |
| **NMO_H_2_S** | 1.64 | 0.008 | 21.0 |
| **NMO_SO_2_** | 3.48 | 0.012 | 15.0 |
| **NMO_NO_2_** | 2.53 | 0.012 | 19.4 |

**Table S3.** The peak-fitting results of Mn 2p high-resolution signal of materials.

| **Samples** | **Assignment** | **E_B_ (eV)** | **FWHM (eV)** | **At. %** |
| --- | --- | --- | --- | --- |
| **NMO** | **Mn 2p_3/2_** _Mn2+_ | 641.1 | 1.1 | 14.1 |
|  | **Mn 2p_3/2_** _Mn3+_ | 642.4 | 1.3 | 42.6 |
|  | **Mn 2p_3/2_** _Mn4+_ | 643.5 | 1.8 | 43.2 |
|  | **Mn 2p_3/2_** _Satellite_ | 645.3 | 2.0 | - |
| **NMO_H_2_O*** | **Mn 2p_3/2_** _Mn2+_ | 640.9 | 1.3 | 17.4 |
|  | **Mn 2p_3/2_** _Mn3+_ | 642.1 | 1.3 | 38.5 |
|  | **Mn 2p_3/2_** _Mn4+_ | 643.2 | 1.8 | 44.2 |
|  | **Mn 2p_3/2_** _Satellite_ | 644.9 | 2.0 | - |
| **NMO_H_2_S** | **Mn 2p_3/2_** _Mn2+_ | 641.2 | 1.3 | 24.0 |
|  | **Mn 2p_3/2_** _Mn3+_ | 642.4 | 1.4 | 38.4 |
|  | **Mn 2p_3/2_** _Mn4+_ | 643.5 | 1.8 | 37.5 |
|  | **Mn 2p_3/2_** _Satellite_ | 645.4 | 2.0 | - |
| **NMO_SO_2_** | **Mn 2p_3/2_** _Mn2+_ | 641.2 | 1.3 | 19.0 |
|  | **Mn 2p_3/2_** _Mn3+_ | 642.4 | 1.8 | 39.8 |
|  | **Mn 2p_3/2_** _Mn4+_ | 643.5 | 1.8 | 41.2 |
|  | **Mn 2p_3/2_** _Satellite_ | 645.2 | 2.0 | - |
| **NMO_NO_2_** | **Mn 2p_3/2_** _Mn2+_ | 641.1 | 1.3 | 15.8 |
|  | **Mn 2p_3/2_** _Mn3+_ | 642.4 | 1.3 | 44.9 |
|  | **Mn 2p_3/2_** _Mn4+_ | 643.5 | 1.7 | 39.3 |
|  | **Mn 2p_3/2_** _Satellite_ | 645.1 | 2.0 | - |

**Table S4.** The peak-fitting results of Mn 3s high-resolution signal of materials.

| **Samples** | **Assignment** | **E_B_ (eV)** | **FWHM (eV)** | **At. %** | **BE_Peak2_ – BE_Peak1_ (eV)*** | **Assignment** |
| --- | --- | --- | --- | --- | --- | --- |
| **NMO** | **Mn 3s** | 84.3 | 3.0 | 66.3 | 4.7 | Mn(III), Mn(IV) |
|  | **Mn 3s** | 89.0 | 3.0 | 33.7 |  |  |
| **NMO_H_2_O*** | **Mn 3s** | 84.5 | 2.9 | 63.9 | 4.6 | Mn(III), Mn(IV) |
|  | **Mn 3s** | 89.1 | 3.0 | 36.2 |  |  |
| **NMO_H_2_S** | **Mn 3s** | 84.1 | 3.2 | 68.9 | 5.0 | Mn(III), Mn(IV) |
|  | **Mn 3s** | 89.1 | 3.0 | 31.1 |  |  |
| **NMO_SO_2_** | **Mn 3s** | 84.5 | 3.0 | 67.9 | 4.7 | Mn(III), Mn(IV) |
|  | **Mn 3s** | 89.1 | 3.2 | 32.1 |  |  |
| **NMO_NO_2_** | **Mn 3s** | 84.4 | 2.9 | 64.5 | 4.6 | Mn(III), Mn(IV) |
|  | **Mn 3s** | 89.0 | 3.5 | 35.5 |  |  |

**Table S5.** The peak-fitting results of O 1s high-resolution signal of materials.

| **Samples** | **Assignment** | **E_B_ (eV)** | **FWHM (eV)** | **At. %** |
| --- | --- | --- | --- | --- |
| **NMO** | **O1s** _O−Na, O−Mn_ | 529.7 | 1.3 | 68.3 |
|  | **O1s** _OH surf_ | 531.4 | 1.6 | 20.3 |
|  | **O1s** _water_ | 533.3 | 1.9 | 11.4 |
|  | **O1s** _Na auger_ | 535.1 | 1.9 | - |
| **NMO_H_2_O*** | **O1s** _O−Na, O−Mn_ | 529.8 | 1.4 | 54.2 |
|  | **O1s** _OH surf_ | 531.7 | 1.8 | 27.6 |
|  | **O1s** _water_ | 532.9 | 1.8 | 18.2 |
|  | **O1s** _Na auger_ | 534.8 | 1.9 | - |
| **NMO_H_2_S** | **O1s** _O−Na, O−Mn_ | 529.7 | 1.3 | 46.0 |
|  | **O1s** _OH surf_ | 531.5 | 1.8 | 45.2 |
|  | **O1s** _water_ | 533.4 | 1.8 | 8.8 |
|  | **O1s** _Na auger_ | 535.5 | 1.9 | - |
| **NMO_SO_2_** | **O1s** _O−Na, O−Mn_ | 529.8 | 1.3 | 65.7 |
|  | **O1s** _OH surf_ | 531.6 | 1.7 | 18.6 |
|  | **O1s** _water_ | 533.0 | 1.8 | 15.7 |
|  | **O1s** _Na auger_ | 534.8 | 1.9 | - |
| **NMO_NO_2_** | **O1s** _O−Na, O−Mn_ | 529.7 | 1.3 | 70.0 |
|  | **O1s** _OH surf_ | 531.4 | 1.6 | 14.0 |
|  | **O1s** _water_ | 532.9 | 1.8 | 16.0 |
|  | **O1s** _Na auger_ | 534.8 | 1.9 | - |

**Table S6.** The peak-fitting results of S 2p high-resolution signal of materials.

| **Samples** | **Assignment** | **E_B_ (eV)** | **FWHM (eV)** | **At. %** |
| --- | --- | --- | --- | --- |
| **NMO_H_2_S** | **S 2p_3/2_** _Sulfide_ | 162.0 | 1.2 | 34.1 |
|  | **S 2p_3/2_** _Sulfur_ | 164.1 | 1.6 | 13.4 |
|  | **S 2p_3/2_** _Sulfate_ | 168.1 | 1.4 | 52.5 |
| **NMO_SO_2_** | **S 2p_3/2_** _Sulfate_ | 168.8 | 1.9 | 100 |

**Table S7.** The peak-fitting results of N 1s high-resolution signal of materials.

| **Samples** | **Assignment** | **E_B_ (eV)** | **FWHM (eV)** | **At. %** |
| --- | --- | --- | --- | --- |
| **NMO_NO_2_** | **N 1s** _Nitrite_ | 403.5 | 1.5 | 32.9 |
|  | **N 1s** _Nitrate_ | 407.0 | 1.4 | 67.1 |
